# Two-sample Mendelian randomization analysis investigates causal associations between gut microbiota and attention deficit hyperactivity disorder

**DOI:** 10.3389/fmicb.2023.1144851

**Published:** 2023-04-24

**Authors:** Lei Wang, Zhihao Xie, Guoliang Li, Guangyao Li, Jianmin Liang

**Affiliations:** ^1^Department of Pediatric Neurology, The First Hospital of Jilin University, Changchun, China; ^2^Jilin Provincial Key Laboratory of Pediatric Neurology, Changchun, China; ^3^The Second Hospital of Jilin University, Changchun, China

**Keywords:** Mendelian randomization, gut microbiota, ADHD, causality, gut-brain axis

## Abstract

Previous research has suggested a link between gut microbiota and attention deficit hyperactivity disorder (ADHD), but their causal relationship has not been elucidated. Aiming to comprehensively investigate their causal relationship and to identify specific causal microbe taxa for ADHD, we conducted a two-sample Mendelian randomization (MR) analysis. Instrumental variables of 211 gut microbiota taxa were obtained from gene wide association study (GWAS), and Mendelian randomization study was carried out to estimate their effects on ADHD risk from PGC GWAS (20,183 ADHD cases and 35,191 controls) and FinnGen GWAS (830 ADHD cases and 215,763 controls). Wald ratio (WR), inverse variance weighted (IVW), MR-Egger, and weighted median were the main methods to analyze causality, and MR results are verified by several sensitivity analysis analyses. At locus-wide significance level (*p* < 1 × 10^−5^), IVW results confirmed that genus *Eubacteriumhalliigroup* (*p* = 0.013) and genus *RuminococcaceaeUCG013* (*p* = 0.049) were correlated with the risk of ADHD and genus *Butyricicoccus* (*p* = 0.009), genus *Roseburia* (*p* = 0.009), genus *Desulfovibrio* (*p* = 0.015), genus *LachnospiraceaeNC2004group* (*p* = 0.026), genus *Romboutsia* (*p* = 0.028) and family *Oxalobacteraceae* (*p* = 0.048) were protective factors of ADHD. Weighted median results indicated that genus *Butyricicoccus* (*p* = 0.018) was negatively correlated with the risk of ADHD. At genome-wide statistical significance level (*p* < 5 × 10^−8^), Wald ratio results demonstrated that genus *Ruminococcustorquesgroup* (*p* = 0.003) was a risk factor for ADHD, while genus *Romboutsia* (*p* = 0.006) and family *Peptostreptococcaceae* (*p* = 0.006) had a negative correlation with the risk of ADHD. In reverse MR analysis, IVW results showed that ADHD may lead to an increase in the abundance of genus *Roseburia* (*p* = 0.020). Analysis of heterogeneity (*p* > 0.05) and pleiotropy (*p* > 0.05) confirmed the robustness of MR results. We demonstrated that there was a potential causal relationship between gut microbiota and ADHD. Our research provides a foundation for understanding the causal relationship between gut microbiota and ADHD, and the several gut bacteria found in this study that may reduce the occurrence of ADHD may have potential in the prevention and treatment of ADHD.

## 1. Introduction

Attention deficit hyperactivity disorder (ADHD) is a neuropsychiatric developmental disease commonly found in children and adolescents. The worldwide prevalence of ADHD among children is estimated to be around 7%. Its core symptoms include inattention, hyperactivity and impulsivity, severely impacting children’s life quality and learning ability (Wolraich et al., 2019). The ADHD pathogenesis and causes of ADHD remain elusive. It is generally recognized that ADHD is influenced both by genetic and environmental risk factors. Studies have revealed that prenatal and postnatal factors (e.g., low birth weight, premature birth) and exposure to environmental toxins (e.g., polychlorinated biphenyls, organophosphate pesticides, and zinc) were common risk factors for ADHD (Sciberras et al., 2017; Posner et al., 2020). In addition, researchers have also found in recent years that the gut microbiota regulated the central nervous system through the microbe-gut-brain axis and participated in the incidence and development of several nervous system diseases (Stilling et al., 2014). Gut microbiota may be associated with multiple neurological diseases such as ADHD (Kalenik et al., 2021), epilepsy (Ding et al., 2021), Alzheimer’s disease (Jiang et al., 2017), and autistic (Hu et al., 2020). Gastrointestinal dysfunction (Ming et al., 2018) and gut microflora dysbiosis (Prehn-Kristensen et al., 2018) are reported in ADHD patients. Furthermore, environmental factors such as perinatal risk factors and diet that play important roles in ADHD also influence gut microbiota composition. For instance, children delivered by cesarean sections will have an increased risk of ADHD compared with vaginal delivery children. Reduced diversity of gut microbiota in cesarean-delivered neonates is related to the risk of ADHD (Talge et al., 2016). Another research indicated that the morbidity of ADHD was lower in breastfed than in non-breastfed children, which might be associated with the changes in the gut microbiota affected by different feeding modes (Pacheco et al., 2015). Pärtty et al. (2015) found that continuous administration of *lactobacillus rhamnosus* GG during the first 6 months of life decreased the risk of ADHD in children. Several randomized controlled trials and cross-sectional studies have indicated that gut microbiota played a critical role in the pathogenesis, prognosis, and treatment of ADHD. However, conventional observational studies are usually susceptible to confounding factors. New methods for investigating the relationship between gut microbiota and ADHD are urgently needed (Xu et al., 2021).

Mendelian randomization (MR) studies estimate the causal effect of a risk factor on an outcome using genetic variants as instrumental variables (Bowden and Holmes, 2019). Benefiting from fruitful findings from large-scale genome-wide association studies (GWASs) at both gut microbiota and disease levels, MR analysis has been widely applied to various scenarios (Xu et al., 2021), including the causal associations between gut microbiota and a variety of neurological diseases. In a recent study, researchers adopt bi-directional MR analysis to assess the causal relationship among the gut microbiota, metabolites, and epilepsy. Four gut microbiota features were identified as potential interventional targets for epilepsy (Ouyang et al., 2022). In addition, the same approach was also applied to judge causal associations between gut microbiota and psychotic disorders such as depression, autism spectrum disorder, and schizophrenia. In a study last year, the relationship between gut microbiota and ADHD was also investigated using MR analysis. However, the study did not support any link between gut bacteria and ADHD (Ni et al., 2021). Given the role of gut microbiota in ADHD is supported by increasing numbers of studies, the above negative result might be associated with the selection of datasets and instrumental variables (IVs).

In this study, we applied the two-sample MR analysis to evaluate the causal relationship between the gut microbiome and ADHD. We hope this research would refresh our understanding of the mutual interaction between gut microbiota and ADHD and be helpful in future investigations.

## 2. Materials and methods

### 2.1. Study design

**Figure 1:**
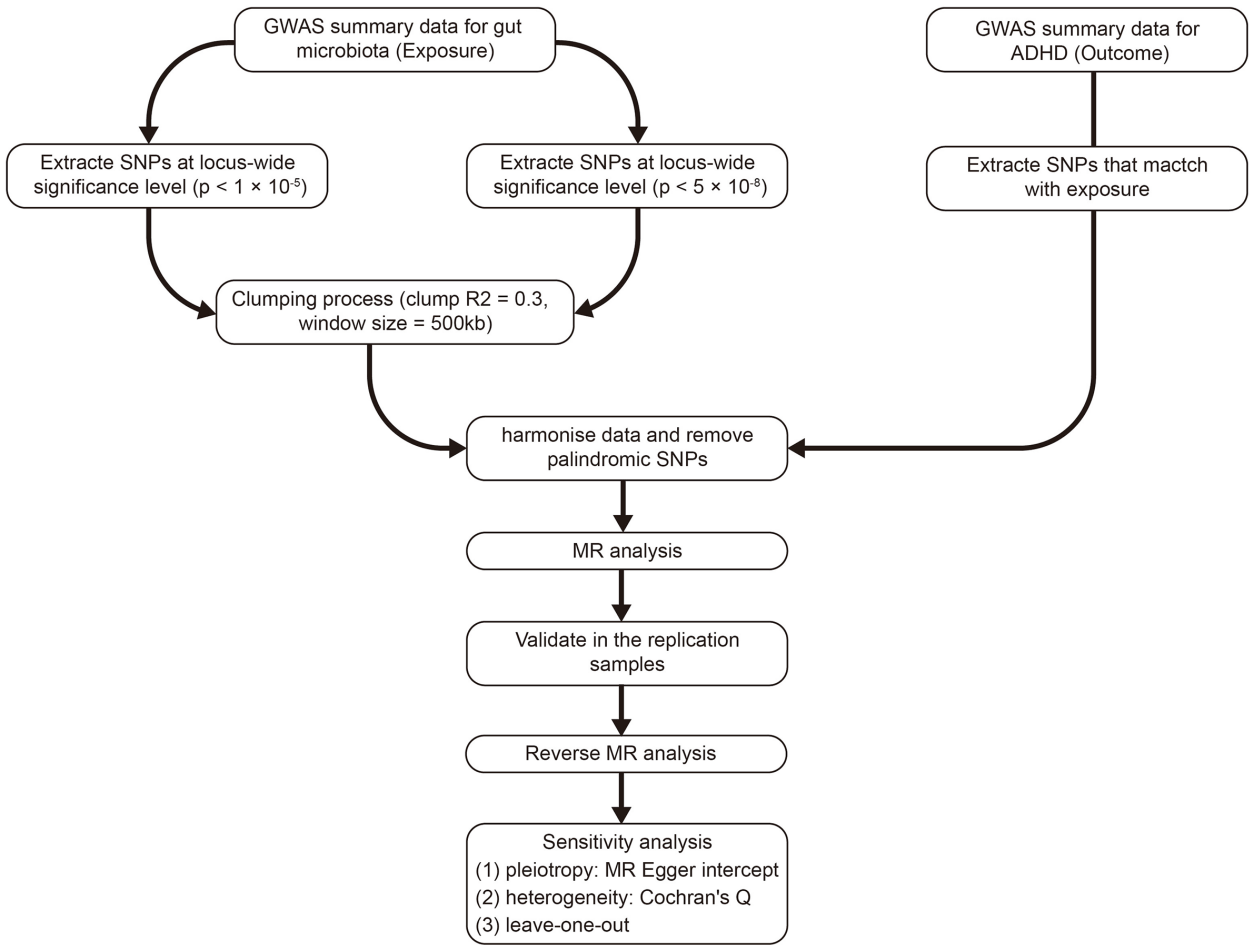
The whole workflow of MR analysis. MR, Mendelian randomization; ADHD, attention deficit hyperactivity disorder.

Figure 1 outlines the study’s overall design. We investigated the association between gut microbiota and the risk of ADHD based on two-sample MR approach.

### 2.2. Ethics statement

Genome-wide association studies summary statistics of both ADHD and microbiota were used for the MR analysis. Each GWAS involved in this study is publicly available through the original research and ethically approved by the respective institutions.

### 2.3. Data sources

Genome-wide association data in gut microbiota were obtained from the MiBioGen study (Kurilshikov et al., 2021), the largest, multiethnic, genome-wide meta-analysis of the gut microbiome to date. The study contains 16S rRNA gene sequencing profiles that target variable regions V4, V3–V4, and V1–V2 of the 16S rRNA gene and whole-genome genotyping data from 18,473 individuals of 25 cohorts, most of whom had European ancestry. The GWAS study eventually yielded 122,110 host genetic variants that were mapped to genetic loci associated with the abundance levels of 211 taxa (9 phyla, 16 classes, 20 orders, 35 families, and 131 genera) by analyzing the gut microbiota taxa variation across different populations.

Genome-wide association studies summary statistics for ADHD were extracted from the Psychiatric Genomics Consortium (PGC) and FinnGen cohort. In the discovery stage, the outcome samples for ADHD were obtained from PGC, including 20,183 cases and 35,191 controls, which identified 12 independent genome-wide loci associated with ADHD. The vast majority of the participants are of European ancestry (18,536 cases and 33,631 controls). All the ADHD cases were diagnosed by psychiatrists at a psychiatric hospital according to ICD10 (F90.0) and genotyped using Illumina PsychChip (Demontis et al., 2019). Replication analyses were performed using the outcome samples from FinnGen. The FinnGen study is a research project that combines genetic data from Finnish biobanks with health records from Finnish health registries. The study uses ICD codes to define the endpoints of the research (Kurki et al., 2022). Our analysis was based on the FinnGen data freeze 5, including 830 cases of ADHD (ICD-10: F90.0) and 215,763 controls. More information about the participants, genotyping and quality control can be found on the FinnGen website.^1^

### 2.4. Two-sample Mendelian randomization

The two-sample Mendelian randomization analysis was performed using the R package “TwoSampleMR” (version 0.5.6). A total of four models were utilized in our Mendelian randomization analysis, including Wald ratio (WR), inverse variance weighted (IVW), MR-Egger, and weighted median. To ensure data robustness and the accuracy of results, the choice of instrumental variables (IVs) included in our models needed to meet three crucial principles: (1) IVs were associated with GM taxa (*p* < 5 × 10^−8^). Due to the small number of eligible IVs (*p* < 5 × 10^−8^), a relatively more comprehensive threshold (*p* < 1 × 10^−5^) was also applied to obtain a more comprehensive result (Liu et al., 2022). (2) There was no association between IVs and ADHD. (3) The IVs were not associated with confounders. Finally, SNPs with linkage disequilibrium were excluded (clump = 500 kb, *r*^2^ = 0.3). In this study, we employed the Wald ratio model for analyzing data when there was only one instrumental variable, and the IVW method was used as the primary analysis method when the number of instrumental variables was two or more. We considered a causal relationship between bacterial taxa and ADHD to exist if the result was significant in the IVW model. Additionally, we used the MR-Egger and weighted median models as references, and if the positive results were replicated in these models, we deemed them to be more robust.

### 2.5. Reverse Mendelian randomization

To investigate whether there is a reverse causal relationship between the screened bacterial taxa and ADHD, we conducted a reverse Mendelian randomization analysis using ADHD as the exposure and the significant bacterial taxa identified in the discovery stage as the outcome. Similar to forward Mendelian randomization, the selection of instrumental variables needs to follow three fundamental principles: (1) IVs were associated with ADHD (*p* < 5 × 10^−8^). (2) There was no association between IVs and bacterial taxa. (3) The IVs were not associated with confounders. Finally, SNPs with linkage disequilibrium were excluded (clump = 500 kb, *r*^2^ = 0.3). We used the inverse variance weighted (IVW) model as the primary analysis model, and if the result was positive in the IVW model, we determined that there was a causal relationship between ADHD and bacterial taxa. We also referred to the results of other models. If the positive result was replicated in the weighted median and MR-Egger models, we considered it to be more robust.

### 2.6. Statistical analysis

In the current study, statistical significance was reported when the *p*-value was less than 0.05 (*p < 0.05). To reduce false-positive results, we performed FDR correction on the *p*-values using the Benjamini–Hochberg method. We computed Cochran’s *Q* statistic to quantify the heterogeneity effects among the selected SNPs. The MR-Egger regression was conducted to assess whether the included SNPs had potential horizontal pleiotropic effects. We also conducted Steiger’s directional test to examine the consistency of the SNP’s effect direction on exposure and outcome. F statistics used to measure the strength of IVs was calculated using the following equation (Papadimitriou et al., 2020):


$$F=R^{2}\left(N-2\right)∕\left(1-R^{2}\right)$$

Where *R*^2^ is the portion of exposure variance explained by the IVs, and N is the sample size. If the number of IVs is less than 10, *R*^2^ is calculated using the following equation:


$$R^{2}=2\times \mathrm{EAF}\times \left(1-\mathrm{EAF}\right)\times {\mathrm{beta}}^{2}$$

Accordingly, R^2^ is calculated using the following equation while the number of IVs is more than 10:


$$R^{2}=\frac{2\times \mathrm{EAF}\times \left(1-\mathrm{EAF}\right)\times {\mathrm{beta}}^{2}}{2\times \mathrm{EAF}\times \left(1-\mathrm{EAF}\right)\times N\times \mathrm{SE}{\left(\mathrm{beta}\right)}^{2}+2\times \mathrm{EAF}\times \left(1-\mathrm{EAF}\right)\times {\mathrm{beta}}^{2}}$$

Where EAF is the effect allele frequency of IV, beta is the effect size of IV on exposure, SE is the standard error of IV on exposure, and N is the number of exposures. It should be noted that this method can only approximate the Explanatory power of the IVs for the exposure, as we do not have genotype data.

## 3. Results

### 3.1. Instrumental variables selection

Supplementary Table S1 shows the presence or absence of corresponding IVs in different bacterial taxa at different stages and thresholds in MR studies. After a series of quality control steps, 1717 unique SNPs (locus-wide significance level, *p* < 1 × 10^−5^) and 18 unique SNPs (genome-wide statistical significance threshold, *p* < 5 × 10^−8^) associated with 211 bacterial taxa were selected as IVs in the discovery stage. In the replication stage, 1937 unique SNPs (*p* < 1 × 10^−5^) and 24 unique SNPs (*p* < 5 × 10^−8^) were selected as IVs (Supplementary Table S2). *F* statistics for IVs fall between 23.944 and 959.585, and all F statistics are greater than 10 (Supplementary Tables S3–S6). The main information of IVs, such as the effect allele, the other allele, beta, SE, and *p-*value, was systematically collected for further analysis.

### 3.2. Two-sample MR analysis

#### 3.2.1. Locus-wide significance level (*p* < 1 × 10^−5^)

**Figure 2:**
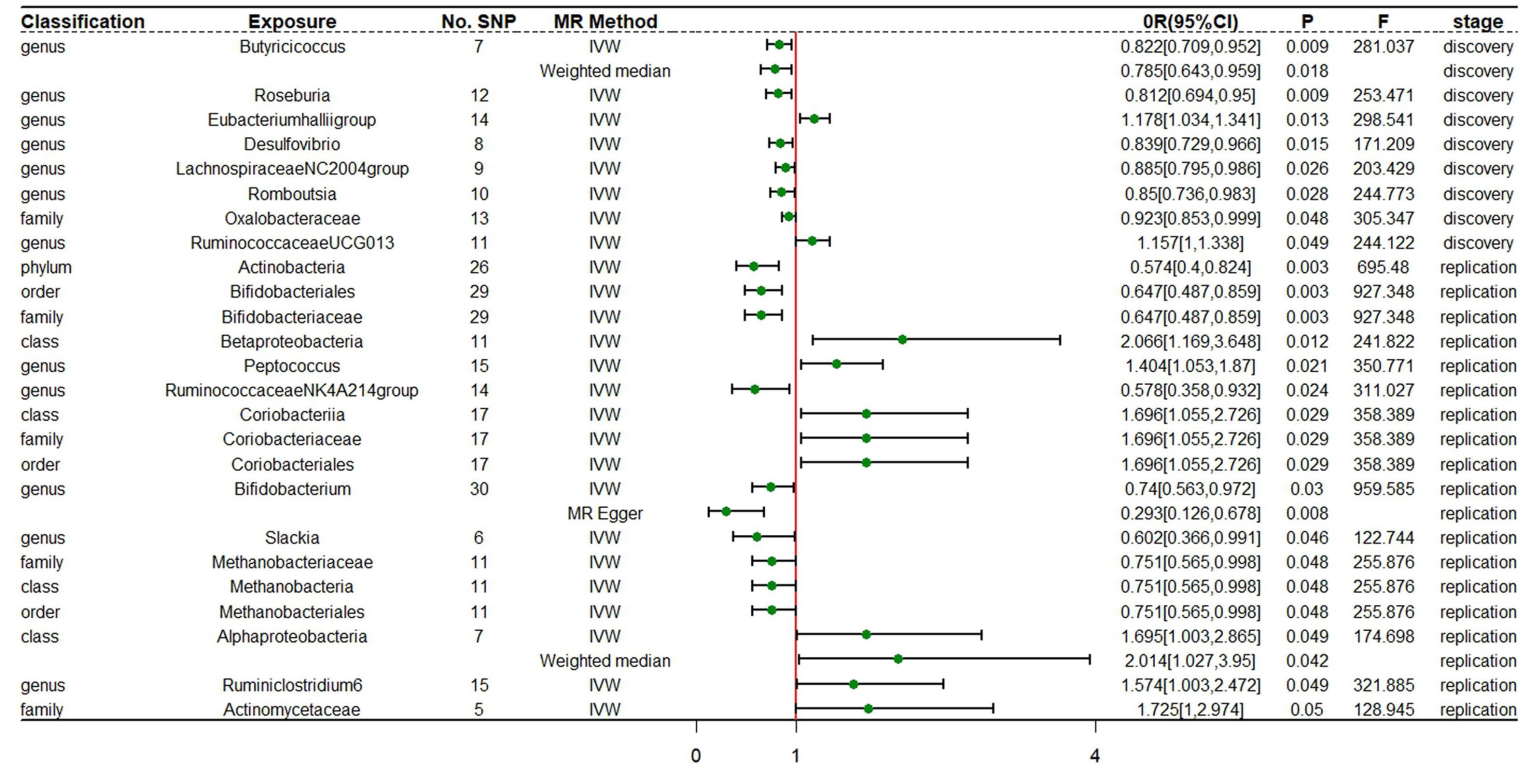
Forest plot of MR estimates at locus-wide significance level (*p* < 1 × 10^−5^). No.SNP, number of SNPs; MR method, the type of model applied in MR analysis; OR, odds ratio; CI, confidence interval; P, *p*-value of causal estimation in different MR methods; F, *F* statistics.

In the discovery stage, the results of IVW analysis demonstrated that at genus level *Eubacteriumhalliigroup* (OR = 1.178, 95% CI 1.034 ~ 1.341, *p* = 0.013) and *RuminococcaceaeUCG013* (OR = 1.157, 95% CI 1 ~ 1.338, *p* = 0.049) were positively correlated with the risk of ADHD and *Butyricicoccus* (OR = 0.822, 95% CI 0.709 ~ 0.952, *p* = 0.009), *Roseburia* (OR = 0.812, 95% CI 0.694 ~ 0.95, p = 0.009), *Desulfovibrio* (OR = 0.839, 95% CI 0.729 ~ 0.966, *p* = 0.015), *LachnospiraceaeNC2004group* (OR = 0.885, 95% CI 0.795 ~ 0.986, *p* = 0.026), *Romboutsia* (OR = 0.85, 95% CI 0.736 ~ 0.983, *p* = 0.028) were negatively correlated with the risk of ADHD. We also found that the family *Oxalobacteraceae* (OR = 0.923, 95% CI 0.853 ~ 0.999, *p* = 0.048) had a negative correlation with the risk of ADHD (Figure 2; Table 1). The MR estimates of the weighted median indicated that *Butyricicoccus* (OR = 0.785, 95% CI 0.643 ~ 0.959, *p* = 0.018) served as a protective factor for ADHD. The detailed statistical results of the 211 intestinal flora samples can be found in Supplementary Tables S1, S2. In sum, eight features (one family and seven genera) were causally associated with ADHD in the discovery sample. Because there are too few significant results after FDR correction, all the above results are screened based on the original *p*-values. However, none of the eight features were able to be reproduced in the replication phase. The complete results of the replication MR analysis can be seen in (Figure 2; Table 2). *Q* statistics of the IVW test and the MR-Egger regression indicated no evidence of heterogeneity and horizontal pleiotropy at the identified results in both discovery and replication samples. Supplementary Tables S3, S4 present the results of MR analysis for all bacterial taxa. The details of each significant MR analysis are shown in Supplementary Figures S1, S2.

**Table 1:** Mendelian randomization (MR) analysis of gut microbiota on ADHD in discovery samples (locus-wide significance level, *p* < 1 × 10^−5^).

Classification	Bacterial taxa (exposure)	No.SNP	MR method	Beta	SE	OR	95% CI	*P*	*F*	Horizontal pleiotropy	Heterogeneity
Genus	Butyricicoccus	7	IVW	−0.197	0.075	0.822	0.709 ~ 0.952	0.009	281.037	0.809	0.581
			Weighted median	−0.242	0.102	0.785	0.643 ~ 0.959	0.018			
Genus	Roseburia	12	IVW	−0.208	0.08	0.812	0.694 ~ 0.95	0.009	253.471	0.911	0.855
Genus	Eubacteriumhalliigroup	14	IVW	0.164	0.066	1.178	1.034 ~ 1.341	0.013	298.541	0.418	0.584
Genus	Desulfovibrio	8	IVW	−0.176	0.072	0.839	0.729 ~ 0.966	0.015	171.209	0.969	0.906
Genus	LachnospiraceaeNC2004group	9	IVW	−0.122	0.055	0.885	0.795 ~ 0.986	0.026	203.429	0.351	0.17
Genus	Romboutsia	10	IVW	−0.162	0.074	0.85	0.736 ~ 0.983	0.028	244.773	0.553	0.502
Family	Oxalobacteraceae	13	IVW	−0.08	0.04	0.923	0.853 ~ 0.999	0.048	305.347	0.434	0.136
Genus	RuminococcaceaeUCG013	11	IVW	0.146	0.074	1.157	1 ~ 1.338	0.049	244.122	0.858	0.156

No.SNP, number of SNPs; MR method, the type of model applied in MR analysis; Beta, estimated causal effect coefficient in different MR methods; SE, standard error of coefficient estimate; OR, odds ratio; CI, confidence interval; P, *p*-value of causal estimation in different MR methods; F, *F* statistics; Horizontal pleiotropy, *p*-value of horizontal pleiotropy analysis; Heterogeneity, *p*-value of heterogeneity analysis.

**Table 2:** Mendelian randomization analysis of gut microbiota on ADHD in replication samples (locus-wide significance level, *P* < 1 × 10^−5^).

Classification	Bacterial taxa (exposure)	No. SNP	MR method	Beta	SE	OR	95% CI	*P*	*F*	Horizontal pleiotropy	Heterogeneity
Phylum	Actinobacteria	26	IVW	−0.554	0.184	0.574	0.4 ~ 0.824	0.003	695.48	0.467	0.75
Order	Bifidobacteriales	29	IVW	−0.436	0.145	0.647	0.487 ~ 0.859	0.003	927.348	0.296	0.119
Family	Bifidobacteriaceae	29	IVW	−0.436	0.145	0.647	0.487 ~ 0.859	0.003	927.348	0.296	0.119
Class	Betaproteobacteria	11	IVW	0.725	0.29	2.066	1.169 ~ 3.648	0.012	241.822	0.25	0.084
Genus	Peptococcus	15	IVW	0.339	0.146	1.404	1.053 ~ 1.87	0.021	350.771	0.667	0.66
Genus	Ruminococcaceae NK4A214 group	14	IVW	−0.549	0.244	0.578	0.358 ~ 0.932	0.024	311.027	0.857	0.972
Class	Coriobacteriia	17	IVW	0.528	0.242	1.696	1.055 ~ 2.726	0.029	358.389	0.69	0.541
Family	Coriobacteriaceae	17	IVW	0.528	0.242	1.696	1.055 ~ 2.726	0.029	358.389	0.69	0.541
Order	Coriobacteriales	17	IVW	0.528	0.242	1.696	1.055 ~ 2.726	0.029	358.389	0.69	0.541
Genus	Bifidobacterium	30	IVW	−0.301	0.139	0.74	0.563 ~ 0.972	0.03	959.585	0.03	0.74
			MR Egger	−1.229	0.428	0.293	0.126 ~ 0.678	0.008			
Genus	Slackia	6	IVW	−0.507	0.254	0.602	0.366 ~ 0.991	0.046	122.744	0.319	0.29
Family	Methanobacteriaceae	11	IVW	−0.286	0.145	0.751	0.565 ~ 0.998	0.048	255.876	0.711	0.102
Class	Methanobacteria	11	IVW	−0.286	0.145	0.751	0.565 ~ 0.998	0.048	255.876	0.711	0.102
Order	Methanobacteriales	11	IVW	−0.286	0.145	0.751	0.565 ~ 0.998	0.048	255.876	0.711	0.102
Class	Alphaproteobacteria	7	IVW	0.528	0.268	1.695	1.003 ~ 2.865	0.049	174.698	0.32	0.634
			Weighted median	0.7	0.344	2.014	1.027 ~ 3.950	0.042			
Genus	Ruminiclostridium6	15	IVW	0.454	0.23	1.574	1.003 ~ 2.472	0.049	321.885	0.99	0.531
Family	Actinomycetaceae	5	IVW	0.545	0.278	1.725	1 ~ 2.974	0.05	128.945	0.912	0.945

No.SNP, number of SNPs; MR method, the type of model applied in MR analysis; Beta, estimated causal effect coefficient in different MR methods; SE, standard error of coefficient estimate; OR, odds ratio; CI, confidence interval; P, *p*-value of causal estimation in different MR methods; F, *F* statistics; Horizontal pleiotropy, *p*-value of horizontal pleiotropy analysis; Heterogeneity, *p*-value of heterogeneity analysis.

#### 3.2.2. Genome-wide statistical significance level (*p* < 5 × 10^−8^)

**Figure 3:**
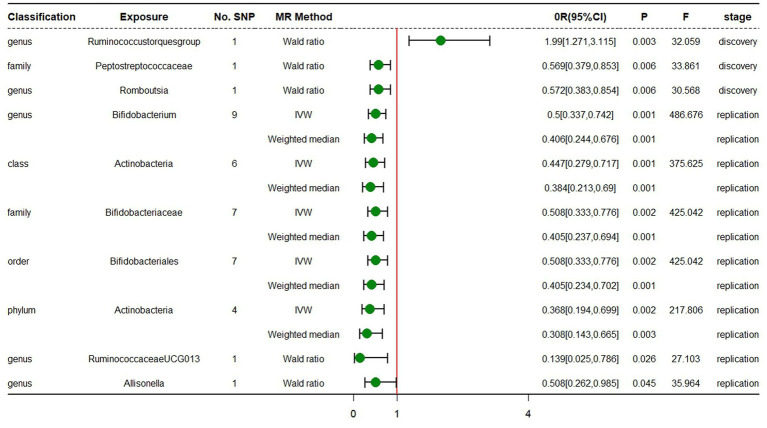
Forest plot of MR estimates at genome-wide statistical significance Level (*p* < 5 × 10^−8^). No.SNP, number of SNPs; MR method, the type of model applied in MR analysis; OR, odds ratio; CI, confidence interval; P, *p*-value of causal estimation in different MR methods; F, F statistics.

In the discovery stage, the results of IVW analysis demonstrated that genus *Ruminococcustorquesgroup* (OR = 1.990, 95% CI 1.271 ~ 3.115, *p* = 0.003) was positively correlated with the risk of ADHD, while genus *Romboutsia* (OR = 0.572, 95% CI 0.383 ~ 0.854, *p* = 0.006) and family *Peptostreptococcaceae* (OR = 0.569, 95% CI 0.379 ~ 0.853, *p* = 0.006) had a negative correlation with the risk of ADHD (Figure 3; Table 3; Supplementary Table S5). All results above are filtered based on the original *p*-values as there are too few significant results after FDR correction. Similarly, we conducted replication analyses using the outcome samples from FinnGen and found that no features were reproduced. The complete results of the replication MR analysis are shown in (Figure 3; Table 4; Supplementary Table S6). MR-Egger regression analysis revealed no horizontal pleiotropy between instrumental variables and outcome. Additionally, the Cochrane *Q* statistics indicated no significant heterogeneity, and the *F* statistics were above 10. The details of each significant MR analysis in the replication stage are shown in Supplementary Figure S3.

**Table 3:** Mendelian randomization analysis of gut microbiota on ADHD in discovery samples (genome-wide statistical significance Level, *p* < 5 × 10^−8^).

Classification	Bacterial taxa (exposure)	No. SNP	MR method	Beta	SE	OR	95% CI	*P*	*F*	Horizontal pleiotropy	Heterogeneity
Genus	Ruminococcus torques group	1	Wald ratio	0.687	0.229	1.990	1.271 ~ 3.115	0.003	32.059		
Family	Peptostreptococcaceae	1	Wald ratio	−0.564	0.207	0.569	0.379 ~ 0.853	0.006	33.861		
Genus	Romboutsia	1	Wald ratio	−0.558	0.205	0.572	0.383 ~ 0.854	0.006	30.568		

No.SNP, number of SNPs; MR method, the type of model applied in MR analysis; Beta, estimated causal effect coefficient in different MR methods; SE, standard error of coefficient estimate; OR, odds ratio; CI, confidence interval; P, *P*-value of causal estimation in different MR methods; F, *F* statistics; Horizontal pleiotropy, *P*-value of horizontal pleiotropy analysis; Heterogeneity, *P*-value of heterogeneity analysis.

**Table 4:** Mendelian randomization analysis of gut microbiota on ADHD in replication samples (genome-wide statistical significance Level, *p* < 5 × 10^−8^).

Classification	Bacterial taxa (exposure)	No. SNP	MR method	Beta	SE	OR	95% CI	*P*	*F*	Horizontal pleiotropy	Heterogeneity
Genus	Bifidobacterium	9	IVW	−0.694	0.201	0.5	0.337 ~ 0.742	0.001	486.676	0.874	0.874
			Weighted median	−0.9	0.259	0.406	0.244 ~ 0.676	0.001			
Class	Actinobacteria	6	IVW	−0.805	0.241	0.447	0.279 ~ 0.717	0.001	375.625	0.945	0.826
			Weighted median	−0.957	0.299	0.384	0.213 ~ 0.69	0.001			
Family	Bifidobacteriaceae	7	IVW	−0.677	0.216	0.508	0.333 ~ 0.776	0.002	425.042	0.616	0.745
			Weighted median	−0.903	0.274	0.405	0.237 ~ 0.694	0.001			
Order	Bifidobacteriales	7	IVW	−0.677	0.216	0.508	0.333 ~ 0.776	0.002	425.042	0.616	0.745
			Weighted median	−0.903	0.281	0.405	0.234 ~ 0.702	0.001			
Phylum	Actinobacteria	4	IVW	−1	0.328	0.368	0.194 ~ 0.699	0.002	217.806	0.613	0.71
			Weighted median	−1.178	0.393	0.308	0.143 ~ 0.665	0.003			
Genus	RuminococcaceaeUCG013	1	Wald ratio	−1.971	0.882	0.139	0.025 ~ 0.786	0.026	27.103		
Genus	Allisonella	1	Wald ratio	−0.678	0.338	0.508	0.262 ~ 0.985	0.045	35.964		

No.SNP, number of SNPs; MR method, the type of model applied in MR analysis; Beta, estimated causal effect coefficient in different MR methods; SE, standard error of coefficient estimate; OR, odds ratio; CI, confidence interval; P, *P*-value of causal estimation in different MR methods; F, *F* statistics; Horizontal pleiotropy, *p*-value of horizontal pleiotropy analysis; Heterogeneity, *p*-value of heterogeneity analysis.

#### 3.2.3. Reverse Mendelian randomization

We performed a reverse Mendelian randomization analysis on 10 gut microbiota taxa (two families and eight genera) identified in the discovery stage. The IVW model showed that the genus *Roseburia* (OR = 1.097, 95% CI 1.015–1.186, *p* = 0.020) had a reverse causal relationship with ADHD, suggesting that ADHD may lead to an increase in the abundance of genus *Roseburia.* MR-Egger regression analysis revealed no horizontal pleiotropy between instrumental variables and outcome. Additionally, the Cochrane *Q* statistics indicated no significant heterogeneity, and the *F* statistics were above 10. The complete results of the reverse MR analysis are shown in (Table 5; Supplementary Table S7; Supplementary Figure S4).

**Table 5:** Reverse MR analysis of ADHD on significant gut microbiota in discovery stage (genome-wide statistical significance Level, *p* < 5 × 10^−8^).

Classification	Bacterial taxa (outcome)	No. SNP	MR method	Beta	SE	OR	95% CI	*P*	*F*	Horizontal pleiotropy	Heterogeneity
Genus	Roseburia	12	IVW	0.093	0.040	1.097	1.015 ~ 1.186	0.020	414.469	0.731	0.079

No.SNP, number of SNPs; MR method, the type of model applied in MR analysis; Beta, estimated causal effect coefficient in different MR methods; SE, standard error of coefficient estimate; OR, odds ratio; CI, confidence interval; P, *P*-value of causal estimation in different MR methods; F, *F* statistics; Horizontal pleiotropy, *P*-value of horizontal pleiotropy analysis; Heterogeneity, *p*-value of heterogeneity analysis.

## 4. Discussion

In this study, we conducted a two-sample MR analysis to investigate the relationship between gut microbiota and ADHD. In the discovery stage, we found that the genus *Eubacteriumhalliigroup*, genus *RuminococcaceaeUCG013*, genus *Butyricicoccus*, genus *Roseburia*, genes *Desulfovibrio*, genus *LachnospiraceaeNC2004group*, genus *Romboutsia*, and family *Oxalobacteraceae* had a causal relationship with ADHD at the locus-wide significance level (*p* < 1 × 10^−5^), while genus *Ruminococcustorquesgroup*, genus *Romboutsia*, and family *Peptostreptococcaceae* had a causal relationship with ADHD at the genome-wide statistical significance level (*p* < 5 × 10^−8^). However, in the replication stage, none of the aforementioned features were replicated. Results of reverse MR analysis showed that genus *Roseburia* had a reverse causal relationship with ADHD.

The term “gut-brain axis” is used to describe the interaction between the gut microbiota and the central nervous system. As part of the gut-brain axis, the gut microbiome influences the symptoms of neurological disorders through metabolic pathways, vagus nerve pathways, and immune pathways. Gut microbiome-produced neurotransmitters such as dopamine, serotonin, and gamma-aminobutyric acid may have an impact on the symptoms of ADHD, according to research (Boonchooduang et al., 2020). Additionally, some bacterial metabolic products such as vitamin B6 and short-chain fatty acids are closely related to ADHD (LeBlanc et al., 2013; Koh et al., 2016). A study led by Tengeler AC discovered that gut microbiota from persons with ADHD could affect the brain in mice (Tengeler et al., 2020). However, it remains controversial whether there are differences in the composition of the gut microbiota between patients with ADHD and healthy controls in observational studies. Some studies have found that there is no significant difference in the diversity of the gut microbiota between ADHD patients and healthy controls (Aarts et al., 2017; Jiang et al., 2018). Other researchers have found that patients with ADHD have reduced alpha diversity of their gut microbiome compared to controls (Prehn-Kristensen et al., 2018). One study also found that children with ADHD had increased alpha diversity compared to non-ADHD controls (Wang et al., 2020). The results of previous studies regarding specific taxonomic microbiota in patients with ADHD are also inconsistent. Prehn-Kristensen et al. (2018) used next-generation sequencing of 16S rDNA to analyze the gut microbiome of 14 patients with ADHD and 17 controls and found that the *Neisseria* and *Bacteroides* genera were increased in adolescents with ADHD. Another study found that the relative abundance of *Bacteroides coprocola* was reduced in the ADHD group compared to healthy controls, while the relative abundance of *Bacteroides uniformis*, *Bacteroides ovatus*, and *Sutterella stercoricanis* increased in the ADHD group (Wang et al., 2020). Cheng et al. (2020) used gene set enrichment analysis (GSEA) to explore the relationship between the gut microbiome and ADHD and found that the genus *Desulfovibrio* and order *Clostridiales* were significantly associated with ADHD. Many studies have suggested a link between changes in the gut microbiota and ADHD, but these studies are often small in scale and lack consensus between studies, making it difficult to draw generalizable conclusions. Additionally, the composition of the gut microbiome may vary in different studies due to differences in gender, race, age, and region. The existence of these uncertain factors hinders the inference of a causal relationship between the gut microbiome and the risk of ADHD.

To more accurately assess the causal relationship between the gut microbiota and ADHD and to control for confounding factors, we conducted a two-sample Mendelian randomization (MR) analysis. Consistent with previous studies, we found that the genus *Roseburia* and genus *Desulfovibrio* were associated with ADHD. Lee et al. (2022) conducted a cross-sectional study that compared the composition of the gut microbiome in 54 drug-naive children with ADHD and 22 healthy controls, and found that the abundance of the phylum *Proteobacteria* and the genera *Roseburia*, *Agathobacter*, *Phascolarctobacterium*, *Prevotella_2*, *Acidaminococcus*, and the *Ruminococcus gnavus group* were increased in the ADHD group compared to the healthy controls. Our study confirms that the increase in genus *Roseburia* abundance is caused by ADHD, but the underlying mechanism remains to be further investigated. GSEA analysis on published GWAS data identified a relationship between the genus *Desulfovibrio* and several psychiatric disorders, including ADHD, autism spectrum disorder (ASD), bipolar disorder, schizophrenia, and major depressive disorder (Cheng et al., 2020). A recent study found that the amount of genus *Desulfovibrio* in the feces of patients with severe autism was significantly higher than in the control group (Finegold et al., 2010). The genus *Roseburia* and genus *Desulfovibrio* seem to be risk factors for ADHD, but our analysis found that they have a protective effect on the development of ADHD. Therefore, further research is needed to understand the role of *Roseburia* and *Desulfovibrio* in the development of ADHD. Another group of gut microbes that may be associated with ADHD is the family *Ruminococcaceae*. Szopinska-Tokov et al. (2020) collected fecal samples from 42 ADHD patients, 15 subthreshold ADHD patients, and 50 healthy controls and found that at the genus level, the relative abundance of *Ruminococcaceae_UCG_003*, *Ruminococcaceae_UCG_004*, *Ruminococcaceae_UCG_005*, *Ruminococcaceae_uncultured*, and *Ruminococcaceae_NK4A214_group* increased in the ADHD participants. The study also found that the relative abundance of *Ruminococcaceae_UCG_004* and *Ruminococcaceae_uncultured* was correlated with symptoms of inattention (Szopinska-Tokov et al., 2020). Dimu Ningshen (DMNS) is a traditional Chinese medicine compound widely used in the clinical treatment of ADHD, Tang et al. (2022) found that DMNS was able to reduce excessive activity in ADHD rats and improve their attention deficit. Additionally, DMNS also reduced the abundance of *Ruminococcaceae_NK4A214_group*, *Eubacterium_nodatum_group*, and *Ruminococcus_2*. The above studies suggest that the family *Ruminococcaceae* may be a risk factor for ADHD. Our study also confirmed that genus *RuminococcaceaeUCG013* is positively correlated with the risk of ADHD. The family *Lachnospiraceae* is another type of gut bacteria that is associated with neurological disorders. Cheng et al. (2020) used the GSEA approach to explore the relationship between gut microbiota and neurological disorders and found that the family *Lachnospiraceae* was associated with major depressive disorder. Another study found that, compared to the control group, ADHD patients had a higher abundance of the genus *Lachnospiraceae UCG-010*. Contrary to this, Wan et al. (2020) compared the differences in bacterial relative abundance in fecal samples from 17 children with ADHD and 17 healthy controls and found a significant decrease in *Lachnospiraceae bacterium* in the ADHD group. Our study indicated that the genus *LachnospiraceaeNC2004group* was a protective factor for ADHD. The specific mechanisms by which the family *Lachnospiraceae* affects the development of ADHD remain to be further studied. A possible conjecture is that the family *Lachnospiraceae* regulates the symptoms of ADHD by producing butyrate. Butyrate has anti-inflammatory, neuroplasticity-promoting, and long-term memory formation effects, and is beneficial for the treatment of neurodegenerative diseases and mental illnesses such as depression and ASD (Stilling et al., 2016). In addition to the genus *LachnospiraceaeNC2004group*, our study also found that there is a causal relationship between bacteria that produce butyrate such as the genus *Roseburia*, genus *Eubacteriumhalliigroup*, and genus *Butyricicoccus* and ADHD (Eicher and Mohajeri, 2022). Although there is currently no research indicating a connection between genus *Eubacteriumhalliigroup*, genus *Butyricicoccus*, and ADHD, genus *Eubacteriumhalliigroup* and genus *Butyricicoccus* seem to have a protective effect in psychiatric disorders such as ASD and Parkinson’s disease. For example, Kong et al. (2021) compared the treatment effects of using probiotics in combination with oxytocin and oxytocin alone in ASD patients and found that patients receiving combination treatment had more pronounced symptom improvement and that the favorable social cognition response of the combination regimen was highly correlated with the abundance of the *Eubacteriumhalliigroup*. However, our study suggests that genus *Eubacteriumhalliigroup* is a risk factor for ADHD. The specific mechanisms by which the genus *Eubacteriumhalliigroup* impacts ADHD need to be further investigated. In recent years, both Bifidobacterium and fecal microbiota transplantation have been explored as potential treatments for ASD in some studies. Abujamel et al. (2022) found that Bifidobacterium and fecal microbiota transplantation significantly increased the abundance of *Butyricicoccus* in a rat model of ASD and improved ASD symptoms. Another study found that the proportion of genus Butyricicoccus was significantly lower in patients with schizophrenia (Ling et al., 2022). Reduction of *Butyricicoccus* has also been observed in patients with postpartum depression (Zhou et al., 2020). Similar to previous research, our MR analysis has for the first time confirmed that genus *Butyricicoccus* may have a protective effect on ADHD. Furthermore, our study also found for the first time a causal relationship between genus *Romboutsia*, family *Oxalobacteracea*, genus *Ruminococcustorquesgroup*, and family *Peptostreptococcaceae* with ADHD. Genus *Romboutsia*, family *Oxalobacteraceae*, and family *Peptostreptococcaceae* are protective factors for ADHD, while genus *Ruminococcustorquesgroup* is a risk factor for ADHD. Almost all of the aforementioned gut bacteria are related to psychiatric disorders in previous studies. For example, compared to healthy controls, the abundance of *Romboutsia* genera is lower in patients with schizophrenia (Yuan et al., 2021). The relative abundance of family *Peptostreptococcaceae* (Fu et al., 2021) and *Ruminococcus torques* (Chen et al., 2022) is relatively increased in ASD patients.

The main advantages of our study include: (1) We analyzed genetic data obtained from a large sample population, making the results more reliable compared to small observational studies. (2) MR analysis prevents the interference of confounders on causal relationships, and the identified causal relationships in our study may provide candidate bacteria for future functional studies. At the same time, some limitations of this study should be fully considered: (1) The GWAS data included in the analysis were mainly from European ancestry subjects, and the results of this study may not apply to other races. (2) 16S rRNA gene sequencing only allows for resolution from the level of phylum to genus, so this study cannot analyze the causal relationship between the gut microbiota and ADHD at a more specific species level. (3) The causal relationship determined in the discovery stage is not replicated in the replication stage. We consider this to be due to the low number of ADHD cases included in the FinnGen study (830 cases and 215,763 controls), which will affect the statistical power of the MR analysis. (4) We cannot determine whether there are overlapping participants in both the exposure and outcome data used in the two sample MR analysis, but bias from participant overlap can be minimized through the application of *F* statistics. (5) Due to significant differences in gut microbiota among children of different ages, an ideal experimental design should include stratified analyses based on age. However, the GWAS data we obtained lacked age information, which limited our ability to explore the causal relationship between gut microbiota and ADHD in different age groups. Nevertheless, the GWAS summary data used in our analysis has been corrected for age, which helped to eliminate the interference of age on the causal relationships we obtained through MR analysis. (6) Studies have shown that ADHD is often accompanied by many comorbidities, including anxiety disorders and behavioral disorders, with prevalence rates as high as 37.9 and 31%, respectively (Mohammadi et al., 2021). Many of these comorbidities have been demonstrated to be associated with the gut microbiota (Socała et al., 2021). Therefore, it is highly likely that there are differences in gut microbiota between individuals with ADHD alone and those with ADHD comorbid with other conditions. Unfortunately, our study was based on existing GWAS data for ADHD, and the original research did not distinguish between ADHD patients and those with comorbidities. Therefore, we were unable to perform a detailed analysis of this issue and further research is necessary to address this limitation. (7) There were essentially no significant results after conducting FDR correction on *p*-values, therefore we had to take the original p-value for screening. However, as an exploratory study, our aim was to identify as many candidate microbial taxa as possible for future research, so the errors introduced by this relatively lenient criterion are acceptable to some extent.

In summary, we evaluated the causal relationship between the gut microbiota and ADHD, using publicly available GWAS summary statistics, and determined specific bacterial groups that may affect the development of ADHD through two-sample MR analysis. Our study provides a foundation for understanding the causal relationship between the gut microbiota and ADHD, and several of the gut bacteria found to potentially reduce the occurrence of ADHD in this study may have potential for use in the prevention and treatment of ADHD. It should be noted that further observational or laboratory-based research is needed to validate these findings. One commonly used approach is to transplant the studied microbial species into germ-free animals to observe their effects. Using this approach, we can transplant specific microorganisms into animal models of ADHD and observe their therapeutic effects. However, the germ-free environment may induce permanent neurodevelopmental defects in the experimental animals, which may interfere with the results (Luczynski et al., 2016). Another common research method involves analyzing the microbiome of fecal samples from patients through microbial sequencing analysis. While traditional 16S rRNA sequencing enables efficient analysis of the composition of microbial communities in the patient’s gut microbiota, its precision is restricted, and we cannot study the microbiota at the species level. Metagenomic sequencing technology, such as shotgun sequencing, enables high-throughput sequencing of the entire genome in a sample. This technology not only allows for more precise identification of the species composition in a sample, but also provides insights into functional composition and metabolic pathways (Gu et al., 2019). Li et al. (2022) utilized this approach to sequence 207 human fecal samples and analyzed the gut microbial profiles of ADHD patients with different phenotypes. All the approaches exemplified above will help us to have a deeper understanding of the role of gut microbiota in ADHD.

## Data availability statement

The original contributions presented in the study are included in the article/Supplementary material, further inquiries can be directed to the corresponding author.

## Author contributions

JML designed the research. LW, GlL, and GyL collected the data. LW analyzed the data and drafted the early version of the manuscript. ZHX collected and organized relevant literature and participated in the subsequent revision of the article. All authors were involved in writing the manuscript and had final approval of the submitted and published versions.

## Funding

This work was supported by the National Natural and Science Foundation of China (Nos. 82271509 and 81771396), the Foundation of Jilin Provincial Key Laboratory of Pediatric Neurology (No. YDZJ202102CXJD021), the Project of Jilin Provincial Science and Technology Development Plan (No. YDZJ202201ZYTS676), and the Project of Jilin Medical and Health Talents (No. JLSWSRCZX2021053).

## Conflict of interest

The authors declare that the research was conducted in the absence of any commercial or financial relationships that could be construed as a potential conflict of interest.

## Publisher’s note

All claims expressed in this article are solely those of the authors and do not necessarily represent those of their affiliated organizations, or those of the publisher, the editors and the reviewers. Any product that may be evaluated in this article, or claim that may be made by its manufacturer, is not guaranteed or endorsed by the publisher.

## Footnotes

^1^ https://finngen.gitbook.io/documentation/
