# Supplementary material for: Two-sample Mendelian randomization analysis investigates causal associations between gut microbiota and attention deficit hyperactivity disorder
Source: Front Microbiol. 2023 Apr 24;14:1144851. doi: 10.3389/fmicb.2023.1144851 (PMC10166206; doi:10.3389/fmicb.2023.1144851)

**Figure legends**

**Supplemental Figure 1. (A) Scatter plots of the association from 8 bacterial features and ADHD in discovery stage at locus-wide significance level (p < 1×10^-5^); Forest plots of causal effects (B) and MR leave-one-out sensitivity analysis (C) for causal associations from 8 bacterial features to ADHD in the IVW test.**

SNP effects were plotted into lines for the inverse-variance weighted test (red line), MR-Egger regression (green line), weighted median estimator (blue line). The slope of the line corresponded to the causal estimation.

**Supplemental Figure 2. (A) Scatter plots of the association from 17 bacterial features and ADHD in replication stage at locus-wide significance level (p < 1×10^-5^); Forest plots of causal effects (B) and MR leave-one-out sensitivity analysis (C) for causal associations from 17 bacterial features to ADHD in the IVW test.**

SNP effects were plotted into lines for the inverse-variance weighted test (red line), MR-Egger regression (green line), weighted median estimator (blue line). The slope of the line corresponded to the causal estimation.

**Supplemental Figure 3. (A) Scatter plots of the association from 5 bacterial features and ADHD in replication stage at genome-wide statistical significance Level (p < 5×10^-8^); Forest plots of causal effects (B) and MR leave-one-out sensitivity analysis (C) for causal associations from 7 bacterial features to ADHD in the IVW test.**

SNP effects were plotted into lines for the inverse-variance weighted test (red line), MR-Egger regression (green line), weighted median estimator (blue line). The slope of the line corresponded to the causal estimation.

**Supplemental Figure 4. (A) Scatter plots of the association from ADHD and** **genus *Roseburia* in reverse MR; Forest plots of causal effects (B) and MR leave-one-out sensitivity analysis (C) for causal associations from ADHD to genus *Roseburia* in the IVW test.**

SNP effects were plotted into lines for the inverse-variance weighted test (red line), MR-Egger regression (green line), weighted median estimator (blue line). The slope of the line corresponded to the causal estimation.

**Supplemental Figure 1**


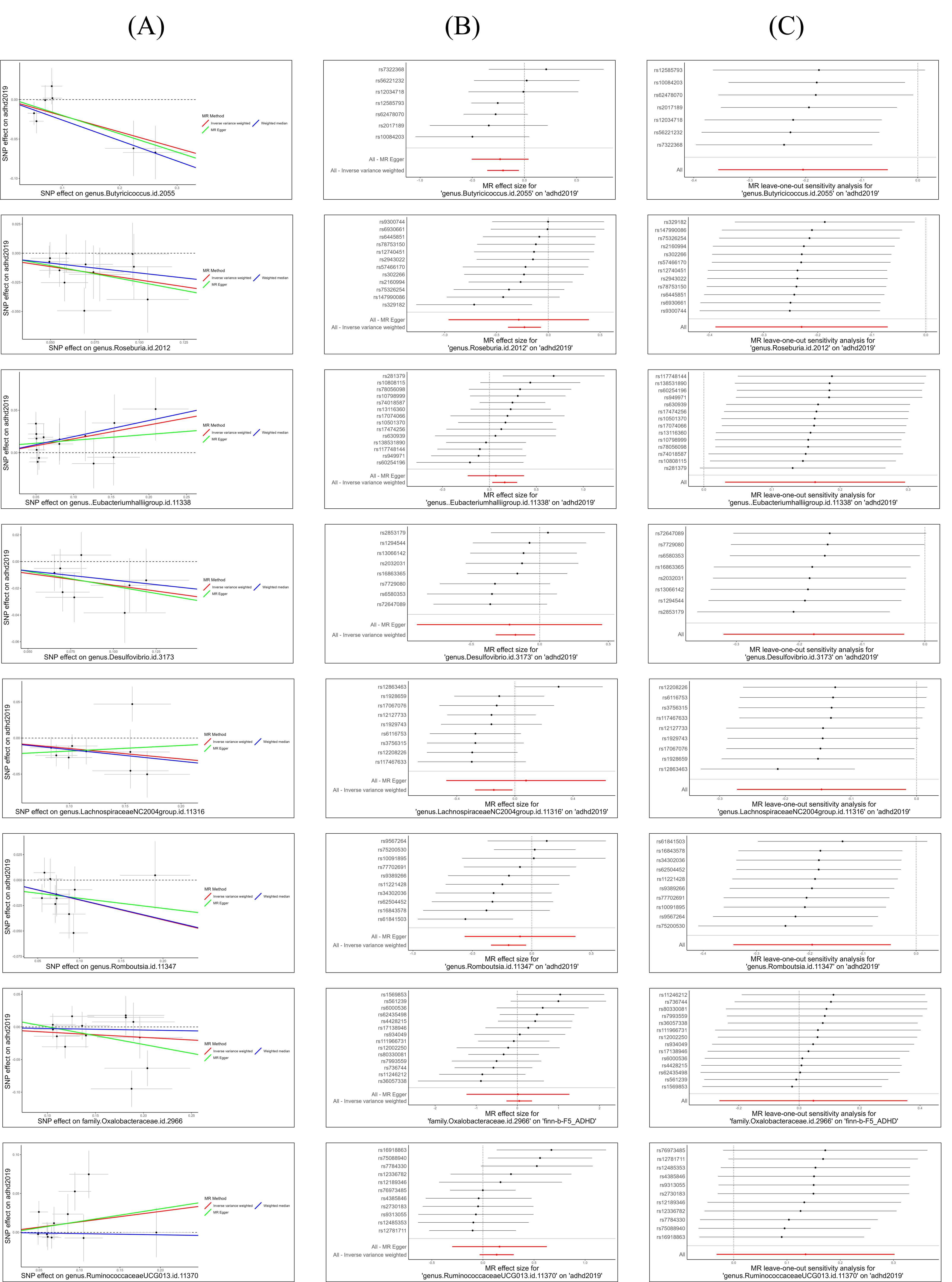


**Supplemental Figure 2**


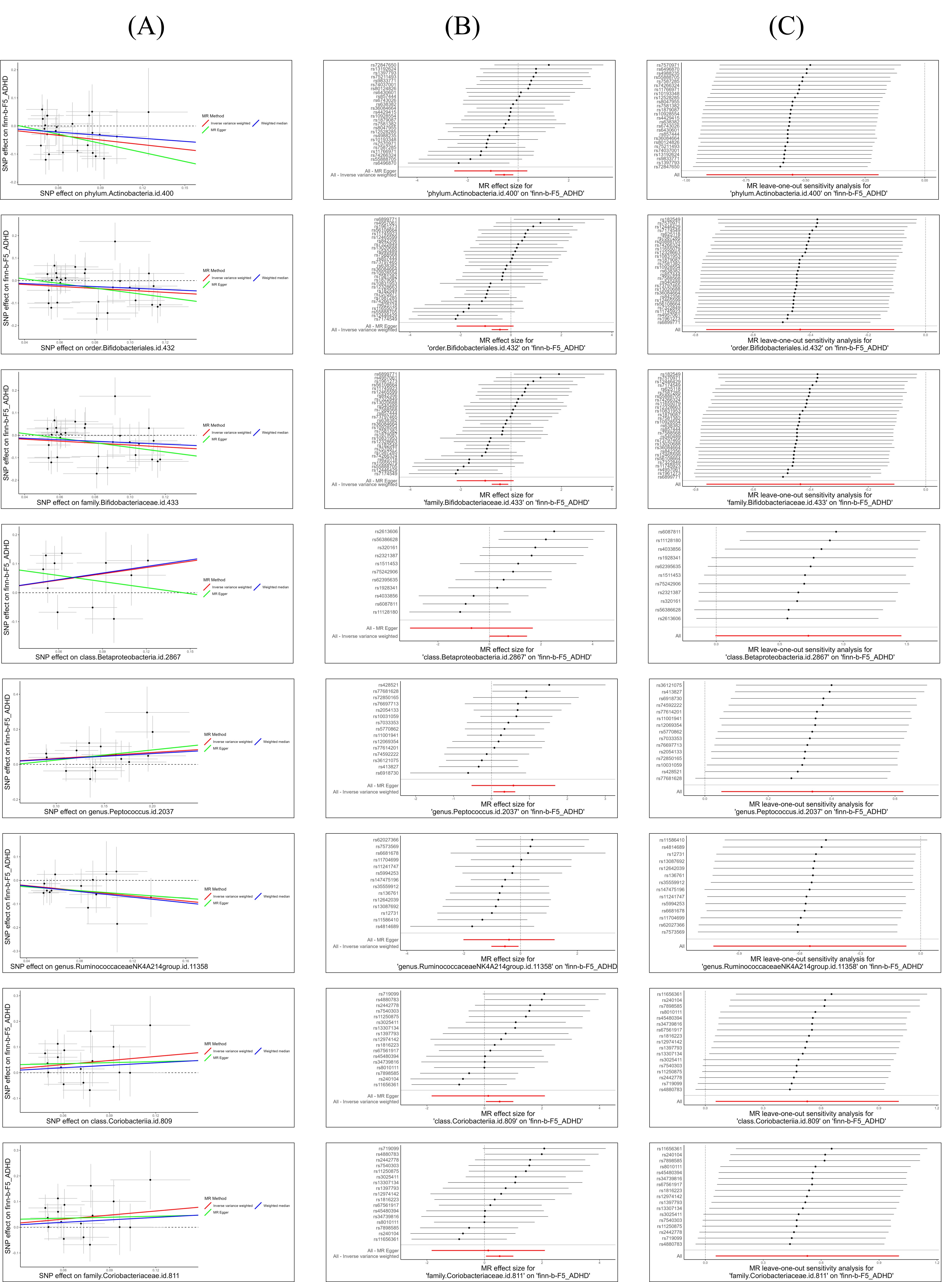


**Supplemental Figure 2 (continued)**


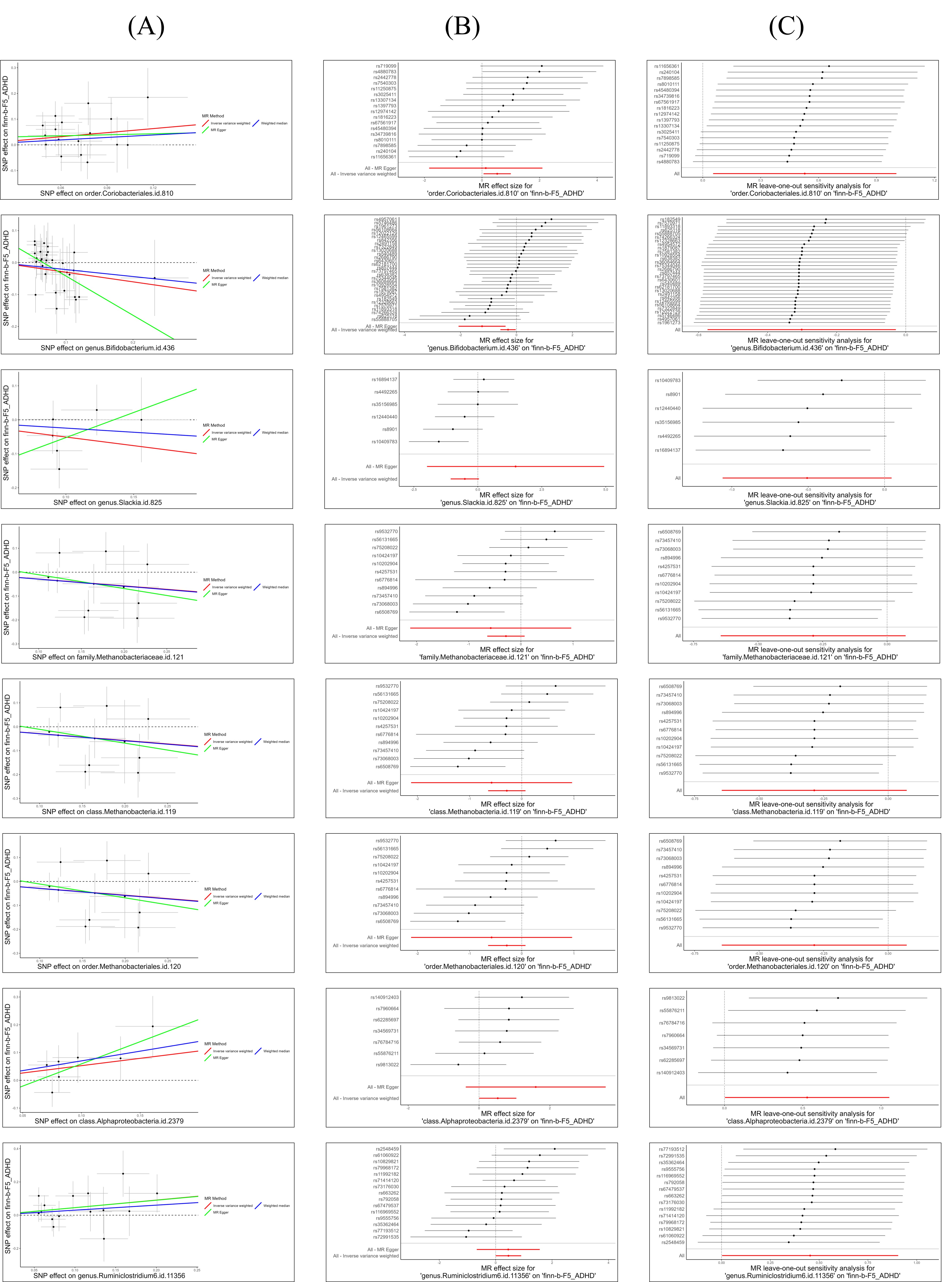


**Supplemental Figure 2 (continued)**


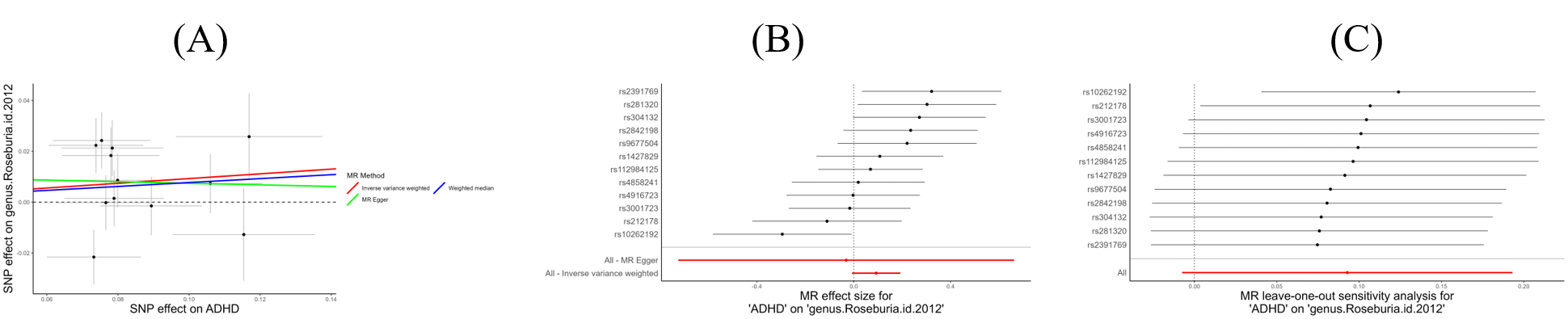


**Supplemental Figure 3**


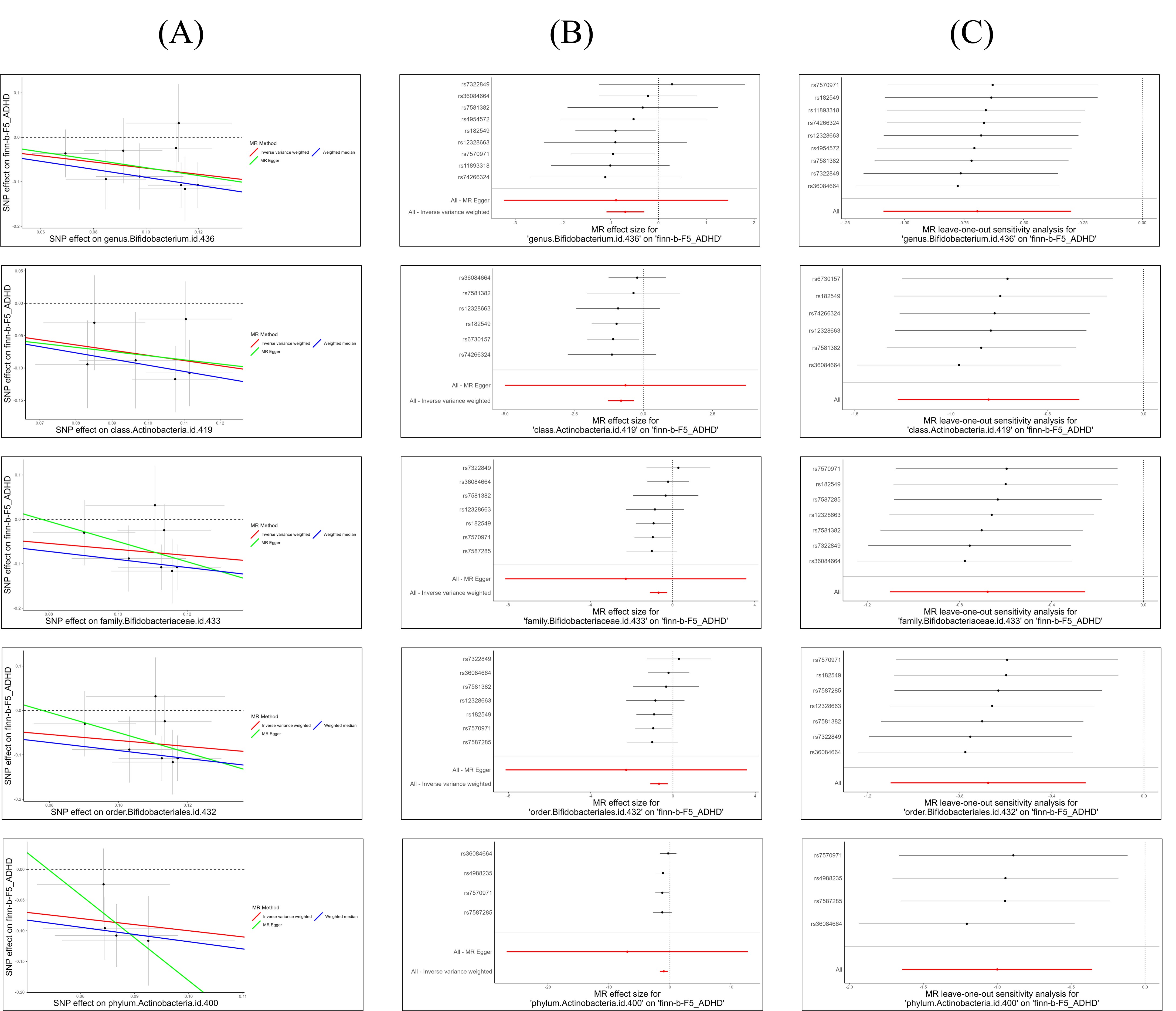


**Supplemental Figure 4**


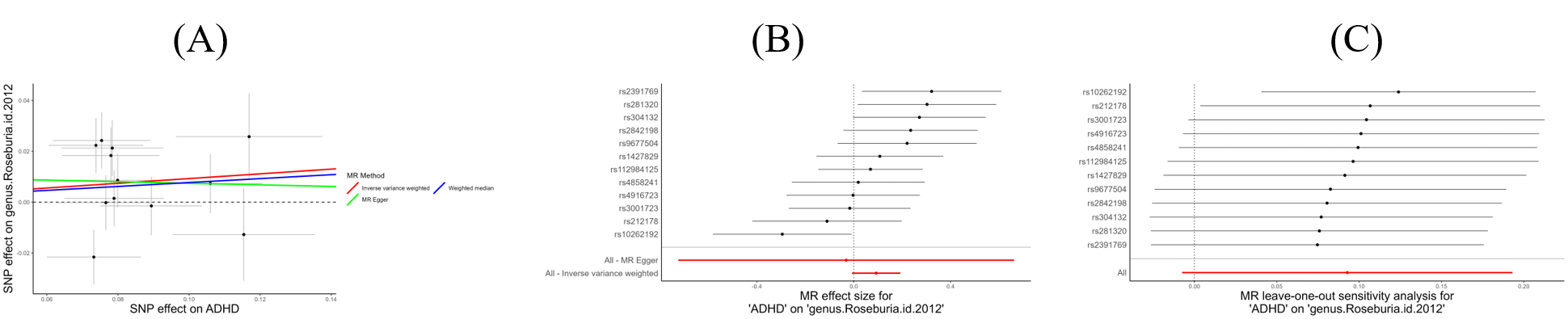

Supplement: Supplementary file 2 [file Table_2.DOCX]
